# Using MemTrax memory test to screen for post-stroke cognitive impairment after ischemic stroke: a cross-sectional study

**DOI:** 10.3389/fnhum.2023.1195220

**Published:** 2023-07-17

**Authors:** Xiaoxiao Zhao, Shujuan Dai, Rong Zhang, Xinjie Chen, Mingjie Zhao, Michael F. Bergeron, Xianbo Zhou, Junyan Zhang, Lianmei Zhong, J. Wesson Ashford, Xiaolei Liu

**Affiliations:** ^1^Department of Neurology, The First Affiliated Hospital of Kunming Medical University, Kunming, China; ^2^Yunnan Province Clinical Research Center for Neurological Disease, Kunming, China; ^3^Department of Neurology, Kunming Second People’s Hospital, Kunming, Yunnan, China; ^4^Department of Neurology, The First Affiliated Hospital of Dali University, Dali, China; ^5^Department of Health Sciences, University of Hartford, West Hartford, CT, United States; ^6^Zhongze Therapeutics, Shanghai, China; ^7^Center for Alzheimer’s Research, Washington Institute of Clinical Research, Vienna, VA, United States; ^8^Department of Clinical Epidemiology and Evidence-based Medicine, Shanxi Bethune Hospital, Shanxi Academy of Medical Sciences, Taiyuan, China; ^9^Bothwin Clinical Study Consultant, Shanghai, China; ^10^War Related Illness and Injury Study Center, VA Palo Alto Health Care System (HCS), Palo Alto, CA, United States; ^11^Department of Psychiatry and Behavioral Sciences, Stanford University, Stanford, CA, United States

**Keywords:** stroke, cognitive impairment, analyses, cross-sectional, executive function, assessment

## Abstract

**Background:**

Whereas the Montreal Cognitive Assessment (MoCA) and Addenbrooke’s cognitive examination-revised (ACE-R) are commonly used tests for the detection of post-stroke cognitive impairment (PSCI), these instruments take 10–30 min to administer and do not assess processing speed, which is a critical impairment in PSCI. MemTrax (MTx) is a continuous recognition test, which evaluates complex information processing, accuracy, speed, and attention, in 2 min.

**Aim:**

To evaluate whether MTx is an effective and practical tool for PSCI assessment.

**Methods:**

This study enrolled acute ischemic stroke (AIS) patients who have assessed the cognitive status including MTx, clinical dementia rating (CDR), MoCA, Neuropsychiatric Inventory (NPI), Hamilton depression scale (HAMD), Hamilton anxiety scale (HAMA), the National Institute of Health Stroke Scale (NIHSS), modified Rankin scale (mRS), and Barthel Index of activity of daily living (BI) combined with the physical examinations of the neurologic system at the 90-day (D90) after the AIS. The primary endpoint of this study was establishing MTx cut-offs for distinguishing PSCI from AIS.

**Results:**

Of the 104 participants, 60 were classified to the PSCI group. The optimized cut-off value of MTx-%C (percent correct) was 78%, with a sensitivity and specificity for detecting PSCI from Non-PSCI of 90.0 and 84.1%, respectively, and an AUC of 0.919. Regarding the MTx-Cp (Composite score = MTx-%C/MTx-RT), using 46.3 as a cut-off value, the sensitivity and specificity for detecting PSCI from Non-PSCI were 80.0 and 93.2%, with an AUC of 0.925. Multivariate linear regression showed that PSCI reduced the MTx-%C (Coef. −14.18, 95% CI −18.41∼−9.95, *p* < 0.001) and prolonged the MTx-RT (response time) (Coef. 0.29, 95% CI 0.16∼0.43, *p* < 0.001) and reduced the MTx-CP (Coef. −19.11, 95% CI −24.29∼−13.93, *p* < 0.001).

**Conclusion:**

MemTrax (MTx) is valid and effective for screening for PSCI among target patients and is a potentially valuable and practical tool in the clinical follow-up, monitoring, and case management of PSCI.

## Introduction

According to the latest report by Global Burden of Diseases (GBD) 2019 Stroke Collaborators, stroke is still one of the two most common causes of death and the third most common cause of death and disability combined (Feigin et al., 2021). The most challenging long-term sequelae affecting stroke survivors include physical, emotional, and cognitive impairments (Intercollegiate Stroke Working Party, 2012).

Post-stroke cognitive impairment (PSCI) is a significant complication and affects over 40% of stroke survivors (Lo et al., 2019). Due to the unlimited variation of brain lesions and diffuse vascular brain injuries among stroke patients, PSCI is heterogeneous (Moore and Demeyere, 2022). Aphasia syndromes arising from lesions in language networks, impairments in memory systems, executive and attention deficits, and visuospatial dysfunction are typical domain-specific impairments in PSCI patients (Corbetta et al., 2005; Jokinen et al., 2015; Dichgans and Leys, 2017). Despite this pervasiveness, there is no easy way to screen for and monitor PSCI other than the traditional paper and pencil cognitive assessment tools (Khaw et al., 2021). Assessment difficulty and heterogeneity of cognitive deficits are likely the major contributing factors to the widespread underdiagnosis of PSCI (Quinn et al., 2021) and are a hindrance to its successful management. The most widely used cognitive assessment tests/scales, such as ACE-R, Informant Questionnaire on Cognitive Decline in the Elderly (IQCODE), and Montreal Cognitive Assessment (MoCA), are time-consuming taking ∼10–30 min to administer, which is manageable in research settings but can be challenging in routine clinical settings. Other extensively utilized tests which take a relatively shorter time to administer, such as the Mini-Cog and mini-mental state examination (MMSE), screen fewer cognitive domains (Bartos and Fayette, 2018). All the above tests have limitations with poor test-retest stability and with the floor effect not being considered for those with lower education (Gottesman and Hillis, 2010). Clinicians need assessment tools that can screen for PSCI sensitively, quickly, and cost-effectively. Furthermore, attention and executive function deficits are two of the major features of PSCI, which processing speed or reaction time is often used to measure (Khaw et al., 2021), but none of the above-mentioned paper and pencil assessment tools measure reaction/response time.

Due to the dynamic and temporal features of the cognitive function changes after stroke, longitudinal and continuous follow-up of cognitive function is recommended for all stroke patients (Rost et al., 2022). For the convenience of physicians and patients, telemedicine has developed widely and effectively in the past decade (Duffy and Lee, 2018). And during the COVID-19 pandemic, patient management and healthcare delivery have faced unique challenges with limits on traditional in-person visits (Hollander and Carr, 2020). Alternatively, online screening and brief, but comprehensive, computerized cognitive assessment are valuable options for tracking cognitive function (Ashford et al., 2022). Therefore, there is an urgent need for a better cognitive screening tool that is simple and easy to implement and has high sensitivity and specificity to screen for PSCI.

We have recently cross-validated a 1.5 to 2-min online digital cognitive assessment tool, the MemTrax (MTx) “continuous recognition task” (Bergeron et al., 2020). MTx specifically assesses information processing and storage in episodic memory, including processing speed via response time, for the estimation of mild cognitive impairment (MCI) associated with early and mild Alzheimer’s disease (AD) (Chen et al., 2021). Based on the well-known features of PSCI (Khaw et al., 2021), we hypothesized that MTx could validly screen for PSCI. Therefore, we designed a study to test the effectiveness and reliability of MTx to distinguish cognitive deficits among patients after ischemic stroke.

## Materials and methods

### Study population

This cross-sectional study recruited participants from an ischemic stroke cohort between January 2020 and December 2020 at the in-patient neurology department of the First Affiliated Hospital of Kunming Medical University. This ischemic stroke cohort baseline data comprising the National Institute of Health Stroke Scale (NIHSS), modified Rankin scale (mRS), Barthel Index of activity of daily living (BI), clinical dementia rating (CDR), Neuropsychiatric Inventory (NPI), Hamilton depression scale (HAMD), and Hamilton anxiety scale (HAMA) were used to check whether the participants qualified to be enrolled in this study. The acute ischemic stroke (AIS) patients who met the enrolling criteria were assessed at the 90-day (D90) post-stroke follow-up. Participants were evaluated using CDR, MoCA, NPI, HAMD, HAMA, NIHSS, mRS, and BI, combined with physical examinations of the neurologic system. Only when they had an MRI-confirmed AIS and met the criteria for vascular cognitive disorders [a VASCOG Statement 2014 (Sachdev et al., 2014)] at D90 were classified into the PSCI group: (i) had risk factors of cerebral vascular disease, (ii) had performance decline in one or more domains which typically occur after ischemic stroke, (iii) the cognitive decline resulted in greater exertion or compensatory strategies to keep independent daily life or the cognitive impairments led to dependency, and (iv) neuro-imaging confirmed the etiology of cognitive decline was due to vascular disease (Revised-Supplementary Figure 1). This is the “golden standard” that distinguishes PSCI from Non-PSCI and determines sensitivity/specificity and ROC. This study was performed according to the Helsinki declaration of 1975 and was reviewed and approved by the ethical committee of the First Affiliated Hospital of Kunming Medical University in Kunming, Yunnan, China (2020-L-56). All participants voluntarily provided signed informed consent.

### Inclusion criteria

1. Diagnosed as AIS according to the Chinese guidelines for diagnosis and treatment of acute ischemic stroke 2018 (Peng and Wu, 2018);
2. age range 18 to 80 years old;
3. willingness to participate in this clinical study and signed informed consent.

### Exclusion criteria

1. Suffering from severe systemic disease(s);
2. intracranial hemorrhagic disorders seen in a cranial CT/MRI;
3. any factor that may have hindered the completion of a neuropsychological assessment;
4. prior cognitive impairment or dementia previously (baseline CDR ≥ 0.5);
5. presence of malignant tumors or undergoing antineoplastic therapy;
6. a history of participating in other clinical studies within 3 months prior to the date of informed consent or currently participating in other clinical studies;
7. clinically significant psychiatric/psychological disease;
8. other situations that may affect full engagement.

### Data collection

A general questionnaire was administered to collect demographic information. The serial neuropsychological assessments (CDR, MoCA, NPI, HAMD, HAMA, NIHSS, mRS, and BI), except MTx, were administered by a qualified and experienced clinician. MTx was the last test for the participants, and it was evaluated by an independent neuropsychologist who was blinded to this study. That is, the evaluator who implemented MTx did not know to which group each subject was classified. MTx scores were recorded on paper for each participant tested. All study data were uploaded into Excel spreadsheets by the researcher who administered the tests. Entries were verified by a colleague before analyses.

### MTx test procedure

The computerized MTx tool^1,^^2^ is a simple and brief continuous recognition assessment that can be self-administered online to measure challenging, timed episodic memory performance. The MTx test procedure has been described in detail previously (Bergeron et al., 2020). Briefly, the test consists of 50 stimuli, 25 unique images (5 items from each of 5 categories of different genres of pictures, culled from a library of 3,000 classified images), and 25 repeated images presented in a pseudo-random order where 4 different order sets were employed. The pictures are chosen for being engaging and visually complex, but the categories are nameable as are some of the features, so left and right brain functions are both reflected in the performance measures, as are sensory and executive cortical functions, which is relevant for non-specific stroke assessment. Notably, over 600 unique tests can be administered in the version of MemTrax used to minimize practice and learning effects. The subject is instructed to respond (touch a screen) as quickly as possible, but only when a repeated image appears, after which the next image is shown immediately. The subject is given up to 3 seconds to respond to each image. Using the internal clock of the local device, the response time (MTx-RT) for each image was determined and recorded for every image by the elapsed time from the presentation of each image to when the screen was touched by the participant (or a full 3 seconds was recorded for each no response). Percent correct (MTx-%C) was calculated to indicate the percentage of repeat images to which the user responded correctly (true positive + true negative divided by 50). The median response time for normal individuals is approximately 0.8 seconds, so the complete test usually takes 95 seconds, while a severely impaired test performance rarely takes more than 120 seconds.

### Statistical analyses

Continuous variables with normal distribution were presented as the mean followed by standard deviation. Variables that were not normally distributed were presented as the median, followed by the first quartile (Q1) ∼ the third quartile (Q3). Student’s *t*-test and Wilcoxon rank-sum test were used for normally and non-normally distributed quantitative data between groups. Categorical variables were analyzed between groups using the Chi-square or Fisher’s exact test. Receiver operating characteristic (ROC) curves were generated to assess the diagnostic accuracy of parameters. The optimized cut-off was computed using Yoden’s index for MTx-%C, MTx-RT, and MTx-Cp (Composite score = MTx-%C/MTx-RT) to compute the sensitivity and specificity for detecting PSCI. The area under the curve (AUC) was used to compare ROC curves. The following calculations were made: positive likelihood ratio (LR+) = sensitivity/(1-specificity); negative likelihood ratio (LR−) = (1-sensitivity)/specificity (Grimes and Schulz, 2005). Multivariable linear regression models were performed to screen the differences of MTx-%C, MTx-RT, and MTx-Cp between the Non-PSCI group and PSCI group. Confounders in which the *p*-value was below 0.1 after univariate analysis were adjusted. Those variates with clinical meanings related to the primary endpoint were adjusted, ignoring the *p*-value. All hypothesis tests were two-sided, and *P* < 0.05 was considered statistically significant. Stata SE 13 (Serial number 401306302851), R software version 3.6.1,^3^ and easy-R^4^ were used for statistical analyses. ROC curves were generated by GraphPad Prism.^5^

The primary endpoint of this study was establishing MTx cut-offs for distinguishing PSCI from AIS. We estimated the sample size based on the area under the ROC curve. Assuming the AUC of 0.8 for the MTx method, which is expected to reach 0.95 in this study, and a type I error of 0.05 with a 90% power, the PASS software calculated the sample sizes of positive and negative cases as 37 participants, respectively.

## Results

### Participant characteristics

**FIGURE 1:**
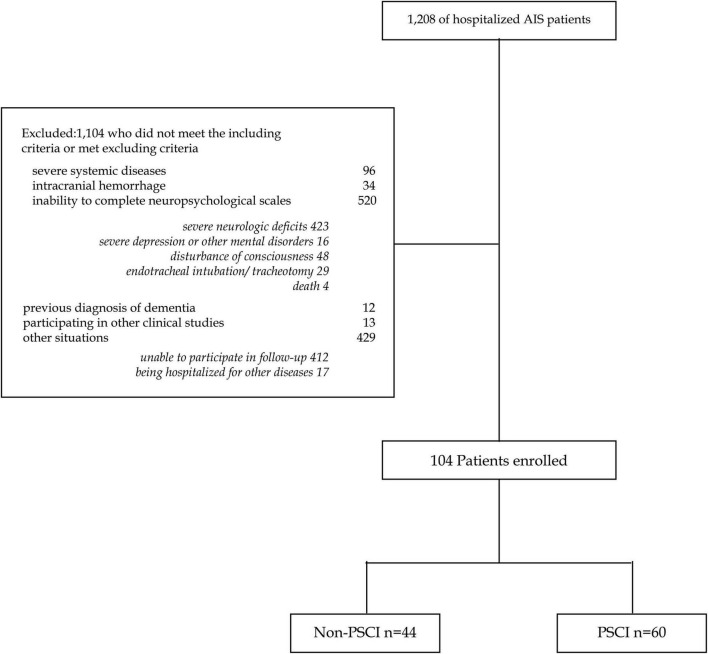
Flow diagram.

Between January 2020 and December 2020, a total of 1,208 AIS patients were hospitalized. After excluding 1,104 patients, 104 participants were enrolled (Figure 1). Analyses were performed on these two groups: (1) Non-PSCI group (*n* = 44); (2) PSCI group (*n* = 60). All participants’ demographic and clinical characteristics are shown in Table 1. The mean age was 62.7 ± 12.7 years old for the Non-PSCI group and 68.8 ± 10.6 years old for the PSCI group. The median education was 12 years in the Non-PSCI group and 7.5 years in the PSCI group. Nineteen participants (43.2%) in the Non-PSCI group were engaged in physical jobs, whereas forty (66.7%) were so engaged in the PSCI group. Regarding clinical characteristics of participants, the two groups had statistically significant differences in hyperhomocysteinemia (13.6% in the Non-PSCI group vs. 31.7% in the PSCI group, *p* = 0.034), typical sleep length (7.6 ± 1.4 h in Non-PSCI group vs. 8.4 ± 1.8 h in PSCI group, *p* = 0.023), the stroke location of AIS (*p* = 0.027), and NIHSS (1.59 ± 1.70 median 1 in Non-PSCI group vs. 2.43 ± 1.99 median 2 in PSCI group, *p* = 0.016). The number of prior cerebral infarctions was not significantly different between the two groups (15.9% more than once in the Non-PSCI group vs. 28.3% in the PSCI group, *p* = 0.137).

**TABLE 1:** Demographic features of the participants.

	Overall (*n* = 104)	Non-PSCI (*n* = 44)	PSCI (*n* = 60)	*t* or *z* or χ^2^	*P*-value
Age (year)					
Mean ± SD	66.21 ± 11.85	62.7 ± 12.7	68.8 ± 10.6	*t* = −2.6591	0.009
Min-max	30–90	30–85	42–90		
Sex (male) (%)	67 (64.4%)	29 (65.9%)	38 (63.3%)	χ^2^ = 0.0735	0.786
Education (y)	9.0 (6.0–13.3)	12.0 (9.0–16.0)	7.5 (5.0–9.5)	*t* = 5.3089	< 0.001
Physical job (%)	59 (56.7%)	19 (43.2%)	40 (66.7%)	χ^2^ = 5.7036	0.017
Right-handed (%)	103 (99.0%)	44 (100.0%)	59 (98.3%)	χ^2^ = 0.7405	0.390
Live alone (%)	4 (3.8%)	3 (6.8%)	1 (1.7%)	χ^2^ = 1.8216	0.177
Take a nap (%)	51 (49.5%)	22 (50.0%)	29 (49.2%)	χ^2^ = 0.0072	0.932
Sleep length (hour)					
Mean ± SD	8.08 ± 1.66	7.6 ± 1.4	8.4 ± 1.8	*t* = −2.3023	0.023
Smoke (%)	49 (47.1%)	23 (52.3%)	26 (43.3%)	χ^2^ = 0.8141	0.367
Alcohol (%)	23 (22.1%)	10 (22.7%)	13 (21.7%)	χ^2^ = 0.0166	0.898
Physical exercise (%)	90 (86.5%)	37 (84.1%)	53 (88.3%)	χ^2^ = 0.3922	0.531
Exercise duration (hour)					
median (IQR)	1.00 (0.50–1.50)	1.0 (0.5–1.5)	1.0 (0.5–1.5)	*z* = −1.319	0.187
Hypertension (%)	75 (72.12%)	31 (70.5%)	44 (73.3%)	χ^2^ = 0.1046	0.746
Diabetes mellitus (%)	24 (23.08%)	11 (25.0%)	13 (21.7%)	χ^2^ = 0.1589	0.690
Brain trauma (%)	4 (3.85%)	2 (4.5%)	2 (3.3%)		1.000
Hypercholesteremia (%)	33 (31.73%)	15 (34.1%)	18 (30.0%)	χ^2^ = 0.1961	0.658
Cerebral hemorrhage (%)	9 (8.65%)	2 (4.5%)	7 (11.7%)	χ^2^ = 1.6285	0.202
Carotid atherosclerosis (%)	80 (76.92%)	31 (70.5%)	49 (81.7%)	χ^2^ = 1.7977	0.180
Atrial fibrillation (%)	6 (5.77%)	1 (2.3%)	5 (8.3%)	χ^2^ = 1.7151	0.190
Coronary heart disease (%)	8 (7.69%)	3 (6.8%)	5 (8.3%)	χ^2^ = 0.0821	0.775
Hyperhomocysteinemia (%)	25 (24.04%)	6 (13.6%)	19 (31.7%)	χ^2^ = 4.5194	0.034
Intravenous thrombolysis (%)	20 (19.23%)	6 (13.6%)	14 (23.3%)	χ^2^ = 1.5367	0.215
Number of cerebral infarction				χ^2^ = 2.2074	0.137
≤ 1	80 (76.92%)	37 (84.1%)	43 (71.7%)		
> 1	24 (23.08%)	7 (15.9%)	17 (28.3%)		
TOAST subtypes				χ^2^ = 4.3248	0.364
CE (%)	7 (6.73%)	2 (4.5%)	5 (8.3%)		
LAA (%)	70 (67.31%)	30 (68.2%)	40 (66.7%)		
OE (%)	3 (2.88%)	2 (4.5%)	1 (1.7%)		
SA (%)	22 (21.15%)	8 (18.2%)	14 (23.3%)		
UE (%)	2 (1.92%)	2 (4.5%)	0 (0.0%)		
Stroke location				χ^2^ = 4.9125	0.027
Anterior circulation (%)	67 (64.42%)	23 (52.3%)	44 (73.3%)		
Posterior circulation (%)	37 (35.58%)	21 (47.7%)	16 (26.7%)		

SD, standard deviation; IQR, interval of quartile range (Q1-Q3); PSCI, post-stroke cognitive impairment group; Non-PSCI, none post-stroke cognitive impairment group; TOAST: Trial of Org 10172 in Acute Stroke Treatment; CE, cardio embolism; LAA, large-artery atherosclerosis; OE, acute stroke of other determined etiology; SA, small-artery occlusion lacunar; UE, stroke of other undetermined etiology; AIS, acute ischemic stroke; NIHSS, National Institute of Health Stroke Scale.

### Differentiating Non-PSCI and PSCI at Day90

MTx-%C was significantly lower in the PSCI group (66.63 ± 9.88) than in the Non-PSCI group (82.16 ± 7.57) (*p* < 0.001). MTx-RT was significantly longer in the PSCI group (1.66 ± 0.32) than in the Non-PSCI group (1.34 ± 0.22) (*p* < 0.001). MTx-Cp was significantly lower in the PSCI group (41.71 ± 10.54) than in the Non-PSCI group (62.98 ± 12.96) (*p* < 0.001). The MoCA corrected scores were significantly different between the PSCI group (18.87 ± 5.85) and the Non-PSCI group (25.80 ± 4.41) (*p* < 0.001). MRS and HAMD were significantly different between the two groups, *p* = 0.043 and *p* = 0.020, respectively, while NIHSS and HAMA were not statistically different between the two groups, *p* = 0.065 and *p* = 0.385, respectively (Table 2).

**TABLE 2:** Clinical assessment results of Non-PSCI and PSCI at D90.

		Non-PSCI (*n* = 44)	PSCI (*n* = 60)	*t* or *z*	*P*-value
**Neuropsychological assessment**					
MTx-%C	Mean ± SD	82.16 ± 7.57	66.63 ± 9.88	*t* = 8.71	< 0.001
MTx-RT	Mean ± SD	1.34 ± 0.22	1.66 ± 0.32	*t* = −5.75	< 0.001
MTx-Cp	Mean ± SD	62.98 ± 12.96	41.71 ± 10.54	*t* = 9.23	< 0.001
MoCA	Mean ± SD	25.80 ± 4.41	18.87 ± 5.85	*t* = 6.60	< 0.001
**CDR *n* (%)**					
0		44 (100)	0		< 0.001 (fisher’s exact)
0.5		0	25 (41.67)		
1		0	22 (36.67)		
2		0	9 (15.00)		
3		0	4 (6.67)		
NPI	Median (IQR)	0 (0–2)	1 (0–6.2)	*z* = −2.73	0.006
HAMA	Median (IQR)	3.00 (2.00–6.25)	4.00 (2.00–8.25)	*z* = −0.87	0.385
HAMD	Median (IQR)	3.00 (0.00–6.00)	3.00 (1.00–9.00)	*z* = −1.28	0.2
**Assessment of neurologic deficits**					
NIHSS	Median (IQR)	0.00 (0.00–1.00)	1.00 (0.00–1.25)	*z* = −1.85	0.065
MRS	Median (IQR)	0.00 (0.00–1.00)	1.00 (0.00–1.00)	*z* = −2.03	0.043
BI	Mean ± SD	98.5 ± 6.2	97.2 ± 6.8	*t* = 1.04	0.301

SD, standard deviation; IQR, interval of quartile range (Q1-Q3); PSCI, post-stroke cognitive impairment group; Non-PSCI, none post-stroke cognitive impairment group; MTx-%C, MemTrax percent correct; MTx-RT, MemTrax response time (seconds); MTx-Cp = MTx-%C/MTx-RT; BI, Barthel Index of activity of daily living; CDR, clinical dementia rating; HAMA, Hamilton anxiety scale; HAMD, Hamilton depression scale; MoCA, Montreal Cognitive Assessment; MRS, modified Rankin scale; NIHSS, National Institute of Health Stroke Scale; NPI, Neuropsychiatric Inventory.

The multivariate linear regression analyses were performed, adjusted for age, sex, education, mental/physical job, living status, sleep length, hyper-homocysteinemia, number of cerebral infarctions, and the differently affected brain regions of the AIS (Table 3). Compared with the Non-PSCI group, the PSCI group had significantly lower MTx-%C (Coef. −14.18, 95% CI −18.41∼−9.95, *p* < 0.001), longer MTx-RT (Coef. 0.29, 95% CI 0.16∼0.43, *p* < 0.001), and lower MTx-Cp (Coef. −19.11, 95% CI −24.29∼−13.93, *p* < 0.001). Moreover, MTx-Cp was also minimized by age above 60 (Coef. −5.39, 95% CI −10.52∼−0.26, *p* = 0.040), female (Coef. −5.13, 95% CI −9.96∼−0.30, *p* = 0.038), and live alone (Coef. −14.61, 95% CI −26.57∼−2.65, *p* = 0.017).

**TABLE 3:** Multivariate linear regression analyses of confounding factors for MTx testing.

	Coef.	SE	*t*	95% CI		*P*-value
				lower	upper	
**MTx-C%**						
Age > 60	−1.65	2.11	−0.78	−5.84	2.54	0.436
Sex (female)	−1.29	1.99	−0.65	−5.23	2.66	0.519
Education (> 6 year)	2.19	2.47	0.89	−2.71	7.10	0.377
Mental job	−2.71	2.04	−1.33	−6.77	1.35	0.188
Live alone	−4.62	4.92	−0.94	−14.40	5.15	0.350
Sleep hours (> 9)	1.02	2.56	0.40	−4.08	6.11	0.692
Homocysteinemia	−1.90	2.35	−0.81	−6.58	2.78	0.422
Diabetics	−2.73	2.31	−1.18	−7.32	1.86	0.240
Hypertension	0.97	2.16	0.45	−3.32	5.27	0.653
Hyperlipemia	1.03	2.05	0.50	−3.04	5.09	0.618
Brain trauma	0.66	5.06	0.13	−9.39	10.71	0.896
Number of cerebral infarctions (> 1)	−0.46	2.31	−0.20	−5.05	4.13	0.843
The differently affected regions of the AIS (posterior)	3.09	2.01	1.53	−0.91	7.08	0.129
PSCI	−14.18	2.13	−6.66	−18.41	−9.95	< 0.001
**MTx-RT**						
Age > 60	0.12	0.07	1.78	−0.01	0.25	0.078
Sex (female)	0.09	0.06	1.41	−0.04	0.21	0.161
Education (> 6 year)	−0.03	0.08	−0.34	−0.18	0.13	0.735
Mental job	0.06	0.06	0.87	−0.07	0.18	0.385
Live alone	0.22	0.15	1.42	−0.09	0.52	0.158
Sleep hours (> 9)	0.01	0.08	0.16	−0.15	0.17	0.873
Homocysteinemia	0.04	0.07	0.48	−0.11	0.18	0.630
Diabetics	−0.03	0.07	−0.35	−0.17	0.12	0.728
Hypertension	0.03	0.07	0.45	−0.10	0.16	0.651
Hyperlipemia	−0.01	0.06	−0.22	−0.14	0.11	0.824
Brain trauma	0.10	0.16	0.62	−0.22	0.41	0.534
Number of cerebral infarctions (> 1)	0.02	0.07	0.29	−0.12	0.16	0.776
The differently affected regions of the AIS (posterior)	0.04	0.06	0.71	−0.08	0.17	0.481
PSCI	0.29	0.07	4.44	0.16	0.43	< 0.001
**MTx-CP**						
Age > 60	−5.39	2.58	−2.09	−10.52	−0.26	0.040
Sex (female)	−5.13	2.43	−2.11	−9.96	−0.30	0.038
Education (> 6 year)	1.68	3.02	0.56	−4.32	7.68	0.578
Mental job	−3.18	2.50	−1.27	−8.15	1.79	0.207
Live alone	−14.61	6.02	−2.43	−26.57	−2.65	0.017
Sleep hours (> 9)	0.72	3.14	0.23	−5.51	6.95	0.819
Homocysteinemia	−3.21	2.88	−1.11	−8.93	2.51	0.268
Diabetics	−1.51	2.83	−0.53	−7.12	4.11	0.596
Hypertension	−1.24	2.65	−0.47	−6.50	4.02	0.641
Hyperlipemia	1.57	2.51	0.63	−3.41	6.55	0.532
**MTx-C%**						
Brain trauma	−4.26	6.19	−0.69	−16.55	8.04	0.493
Number of cerebral infarctions (> 1)	−1.84	2.83	−0.65	−7.46	3.78	0.518
The differently affected regions of the AIS (posterior)	0.74	2.46	0.30	−4.15	5.63	0.765
PSCI	−19.11	2.61	−7.33	−24.29	−13.93	< 0.001

Coef, co-efficiency; CI, confidential interval; MTx-%C, MemTrax percent correct; MTx-RT, MemTrax response time (seconds); MTx-Cp = MTx-%C/MTx-RT; PSCI, post-stroke cognitive impairment; AIS, acute ischemic stroke.

### Screening accuracy of MemTrax

**FIGURE 2:**
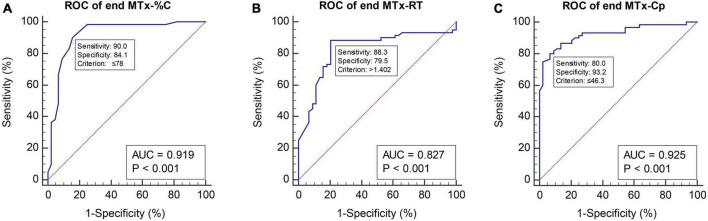
ROC curves of the three metrics of MemTrax. **(A)** ROC curve of end MTx-%C with cutoff point at 78. **(B)** ROC curve of end MTx-RT with cutoff point at 1.402. **(C)** ROC curve of end MTx-Cp with cutoff point at 46.3. MTx-%C, MemTrax percent correct; MTx-RT, MemTrax response time (seconds); MTx-Cp, MemTrax composite score, was derived by multiplying the numbers in MTx-%C and the reciprocal of MTx-RT (= MTx-%C/MTx-RT); ROC, receiver operating characteristic; AUC, area under the curve.

To determine the screening accuracy of MTx, ROC curve analyses were conducted (Figure 2). When the Non-PSCI and PSCI groups were compared, the AUCs were 0.919 (95% CI 0.849∼0.964) for MTx-%C, 0.827 (95% CI 0.741∼0.894) for MTx-RT, and 0.925 (95% CI 0.857∼0.968) for MTx-Cp.

Sensitivity and specificity analyses for different optimized cut-off values of the MTx metrics are shown in Figure 2 and Supplementary Table 1. When using MTx-%C to compare the Non-PSCI and PSCI groups, the optimized cut-off value, which maximized true positives while minimizing false positives, was 78%, with the sensitivity and specificity of detecting PSCI 90.0 and 84.1%, respectively. Further, LR+ and LR− were 5.66 and 0.12, respectively. Using 1.402 s as an optimized cut-off value for MTx-RT, the sensitivity and specificity of detecting PSCI were 88.3 and 79.6%, respectively, while LR+ and LR− were 4.32 and 0.15. Using 46.3 as a cut-off value for MTx-Cp, the sensitivity and specificity of detecting PSCI from Non-PSCI were 80.0 and 93.2%. LR+ and LR− were 11.73 and 0.21, respectively. Such cut-off values were confirmed by logistic regression models which are shown in Supplementary Table 2. Please check Supplementary Figure 2 and Supplementary Tables 3–5 for more detailed information.

## Discussion

In this cross-sectional study based on a post-stroke cohort, we demonstrated the clinical utility of MTx in screening PSCI in patients with ischemic stroke. It complements earlier research findings on mild cognitive impairment and dementia due to AD or vascular dementia in a group of Chinese participants where cognitive impairment and severity were detected (Chen et al., 2021; Liu et al., 2021).

Our previous study on the utility of MTx in AD recommended MTx-%C as the best metric to screen for MCI, with the optimal MTx-%C cut-off being 81% (Liu et al., 2021). In this study, the optimized cut-off value was < 78%, with sensitivity and specificity of detecting PSCI, at 90.0 and 84.1%, respectively. Further, our current study found that both MTx-RT and MTx-Cp are also valuable metrics when screening for PSCI.

A positive LR of 10 or more suggests that a positive test will be very good at ruling the disorder in. A negative LR of 0.1 or less suggests that a negative test will be very good at ruling the disorder out (Davidson, 2002). In our findings, 46.3% as a cut-off value for MTx-Cp had the highest LR+ of 11.73, which means those whose MTx-Cp was lower than this cut-off point might be considered to have PSCI. Meanwhile, when 78% was used as the cut-off value of MTx-%C, LR− was 0.12, which means those whose MTx-%C was greater than this cutoff point might not be considered to have PSCI. Thus, it is recommended to apply MTx-%C to assist in ruling out PSCI, whereas MTx-Cp can be more effective to screen for PSCI at the follow-up points.

Working memory impairment is found in 75% of stroke survivors (Riepe et al., 2004), and additional impairment in executive function is common in patients after stroke (Chung et al., 2013). Attentional deficits are commonly observed within the acute phase and at discharge from the hospital (Loetscher et al., 2019). Cognitive slowing is a common complaint after stroke (Cumming et al., 2013), and the processing speed of information can also be impaired in 20 to 50% of stroke survivors for years (Barker-Collo et al., 2010). Thus, in clinical practice, cognitive assessment to screen for PSCI should include the following five core domains: executive function, attention, memory, language, and visuospatial function (Iadecola et al., 2019). Previous research showed that both the MTx-%C and MTx-RT gave reasonably accurate estimates of the MoCA score. Nevertheless, the MemTrax scores also related highly to the subscores of the MoCA, indicating the breadth of relevance to the cognitive domains (van der Hoek et al., 2019). Based on its design, MTx performance depends on the functioning of these cognitive domains, and its impairment non-specifically reflects dysfunction in these areas. While the primary MTx output metrics primarily assess performance in recognition memory (MTx-%C) and reaction/response time (MTx-RT), these measures are also affected by attention/executive function, as well as visuospatial and motor function. Our findings of lower MTx-%C and longer MTx-RT in the PSCI group compared to those in the Non-PSCI lend support to our hypothesis that the MTx test would be a useful and practical tool for detecting and monitoring PSCI.

Findings in our present study are consistent with reported cognitive impairment incidence of 25–75% in stroke victims (Patel et al., 2002), which results in poorer recovery, more significant functional impairment, increased stroke recurrence, and mortality (Swartz et al., 2016; Rost et al., 2022). The cognitive impairment may occur at the very beginning of the post-stroke period (McClure et al., 2012), or it may appear after 3 months (Patel et al., 2002), 1, 2, or 3 years following the stroke event (Patel et al., 2003). Therefore, detecting and providing therapy for cognitive impairment is crucial in the long-term follow-up and welfare of stroke survivors. These observations suggest that an extended follow-up plan for stroke patients with simple, feasible approaches to routinely screen for PSCI is vitally important (Swartz et al., 2016). Two widely used tools to assess cognition, MoCA and ACE-R, require face-to-face administration and a comparatively long time burden to the patient and healthcare provider, with questions of test-retest validity, learning, and stability (van der Hoek et al., 2019). Accordingly, MTx provides an advantageous and viable alternative as a screening tool to identify and initially select patients for a more comprehensive cognitive assessment.

The COVID-19 pandemic prompted an unintended though necessary widespread transformation to digital and virtual healthcare services (Hachinski, 2022). And with a recognizably prevalent unsatisfactory provider follow-up rate combined with the broad impact and related restrictions resulting from the pandemic (Cadilhac et al., 2022), a remote digital tool that can readily and validly evaluate cognitive status would be an effective solution to monitor complications of post-stroke patients. The 2-min MTx memory test has demonstrated advantages for this population, including the capacity to connect patients to providers more regularly and meet the needs of cognitive screening for PSCI. Furthermore, due to its large sets of unique tests, MTx can be repeatedly used at a regular frequency without compromising accuracy in differentiating cognitive health or losing the interest of patients to perform repeated tests. There are an increasing number of clinical trials focusing on the prevention or treatment of PSCI, such as secondary prevention (Yaffe, 2019), pharmaceutical interventions (Lim et al., 2021), or non-pharmaceutical treatments (Yun et al., 2015). MTx may also be useful for tracking post-stroke cognitive status during the recovery period to assist in determining if the cognitive impairment trend is improving, stable, or deteriorating, as well as evaluating treatment efficacy. In the future, we will explore the value of MTx in predicting PSCI through a cohort study.

The current study has several limitations. First, the sample size was small. Therefore, these results may be viewed as only preliminary, pending confirmation from further larger studies. Additional research focused on a larger group of different patients should be carried out to verify the findings from this study. Second, in this study, the NIHSS scores of patients who voluntarily returned for follow-up were relatively mild, and the screening point was only at D90. In the next research phase, we will include more patients with severe NIHSS scores and extend the follow-up period. Third, this is a cross-sectional study. Future efforts should include implementing a prospective longitudinal study to evaluate stroke patients across different time periods to evaluate the flexibility and effectiveness of MTx for tracking changes of cognitive function. MTx is currently under continuous development. With further comprehensive analyses from MTx to date and ongoing usage in cognitive screening, we anticipate there will be informed updates and variations to the MTx paradigm that will support multi-dimensional testing and assessments to cover broader aspects of cognitive function.

## Conclusion

Our study proved MemTrax was effective in screening for PSCI in AIS patients. This user-friendly computerized could be valuable for managing post-stroke patients, particularly in detecting and monitoring PSCI. Future studies will evaluate MemTrax’s utility in tracking cognitive health and assessing the effectiveness of interventions in post-stroke patients.

## Data availability statement

The original contributions presented in this study are included in the article/Supplementary material, further inquiries can be directed to the corresponding authors.

## Ethics statement

This study was performed according to the Helsinki declaration of 1975 and was approved by the Ethical Committee of the First Affiliated Hospital of Kunming Medical University in Kunming, Yunnan, China (2020-L-56). All participants voluntarily signed an informed consent document.

## Author contributions

XL, JZ, and JA designed study. SD, XZha, and XL wrote the main manuscript text. XZha, RZ, XC, and MZ collected data. JZ and LZ analyzed data. SD, XZha, and JZ prepared figures and tables. MB, JA, XL, and XZho edited the manuscript. All authors contributed to the article and approved the submitted version.

## Footnotes

1
www.memtrax.com

2
www.memtrax.com.cn

3
http://cran.r-project.org/

4
www.empowerstats.com

5
http://www.graphpad-prism.cn
