# Supplementary material for: Using MemTrax memory test to screen for post-stroke cognitive impairment after ischemic stroke: a cross-sectional study
Source: Front Hum Neurosci. 2023 Jul 17;17:1195220. doi: 10.3389/fnhum.2023.1195220 (PMC10387538; doi:10.3389/fnhum.2023.1195220)
Supplement: Supplementary file 1 [file Data_Sheet_1.docx]

# Supplement Document

## Table 1. Sensitivity and specificity analyses for different cut-off values of MemTrax metrics for PSCI outcome

| Cut-off | Sensitivity(%) | 95% CI | Specificity(%) | 95% CI | +LR | -LR |
| --- | --- | --- | --- | --- | --- | --- |
| MTx-%C |  |  |  |  |  |  |
| ≤76 | 83.3 | 71.5 - 91.7 | 86.4 | 72.6 - 94.8 | 6.11 | 0.19 |
| ≤78 | 90.0 | 79.5 - 96.2 | 84.1 | 69.9 - 93.4 | 5.66 | 0.12 |
| ≤80 | 98.3 | 91.1 - 100.0 | 75.0 | 59.7 - 86.8 | 3.93 | 0.022 |
| MTx-RT |  |  |  |  |  |  |
| ≤1.402 | 88.3 | 77.4 - 95.2 | 79.6 | 64.7 - 90.2 | 4.32 | 0.15 |
| ≤1.500 | 73.3 | 60.3 - 83.9 | 79.6 | 64.7 - 90.2 | 3.59 | 0.34 |
| ≤1.152 | 73.3 | 60.3 - 83.9 | 81.8 | 67.3 - 91.8 | 4.03 | 0.33 |
| MTx-Cp |  |  |  |  |  |  |
| ≤45.6 | 76.67 | 64.0 - 86.6 | 93.18 | 81.3 - 98.6 | 11.24 | 0.25 |
| ≤46.3 | 80.00 | 67.7 - 89.2 | 93.18 | 81.3 - 98.6 | 11.73 | 0.21 |
| ≤46.9 | 80.00 | 67.7 - 89.2 | 90.91 | 78.3 - 97.5 | 8.80 | 0.22 |

CI, confidential interval; MTx-%C, MemTrax percent correct; LR+, positive likelihood ratio; LR-, negative likelihood ratio; MTx-RT, MemTrax response time (seconds); MTx-Cp=MTx-%C/MTx-RT

## Table 2. Univariate analysis for the risk factor of PSCI

|  | OR(95%CI) | p-value |
| --- | --- | --- |
| Age (>60) | 1.7 (0.8, 4.0) | 0.197 |
| Female | 1.1 (0.5, 2.5) | 0.786 |
| Education years (> 6) | 0.2 (0.1, 0.5) | <0.001 |
| Mental job | 0.4 (0.2, 0.8) | 0.018 |
| Live alone | 4.3 (0.4, 43.0) | 0.212 |
| Sleep hours (>9) | 4.2 (1.1, 15.5) | 0.034 |
| Homocysteinemia | 2.9 (1.1, 8.1) | 0.038 |
| Diabetics | 0.8 (0.3, 2.1) | 0.690 |
| Hypertension | 1.2 (0.5, 2.7) | 0.746 |
| Hyperlipemia | 0.8 (0.4, 1.9) | 0.658 |
| Brain Trauma | 0.7 (0.1, 5.3) | 0.752 |
| Number of cerebral infarctions (>1) | 2.1 (0.8, 5.6) | 0.142 |
| The differently affected regions of the AIS(Posterior) | 0.4 (0.2, 0.9) | 0.028 |
| MTx-%C ≤78 | 47.6 (14.8, 153.0) | <0.001 |
| MTx-RT >1.500 | 10.7 (4.2, 27.1) | <0.001 |
| MTx-Cp ≤46.3 | 25.3 (8.9, 71.8) | <0.001 |

OR, odds ratio; CI, confidential interval; MTx-%C, MemTrax percent correct; MTx-RT, MemTrax response time (seconds); MTx-Cp=MTx-%C/MTx-RT;

## Table 3. Multivariate analysis for the risk factor of PSCI

|  | OR | SE | z | 95% CI | | p-value |
| --- | --- | --- | --- | --- | --- | --- |
|  |  |  |  | Lower | upper |  |
| Model for MTx-%C | | | | | | |
| Age (>60) | 0.73 | 0.58 | -0.40 | 0.15 | 3.51 | 0.690 |
| Education years (> 6) | 0.31 | 0.27 | -1.37 | 0.06 | 1.67 | 0.172 |
| Mental job | 0.59 | 0.40 | -0.77 | 0.15 | 2.26 | 0.440 |
| Sleep hours (>9) | 5.17 | 5.13 | 1.65 | 0.74 | 36.18 | 0.098 |
| Homocysteinemia | 2.06 | 1.78 | 0.84 | 0.38 | 11.22 | 0.402 |
| Number of cerebral infarctions (>1) | 4.33 | 3.97 | 1.60 | 0.72 | 26.14 | 0.110 |
| The differently affected regions of the AIS(Posterior) | 0.72 | 0.50 | -0.48 | 0.19 | 2.78 | 0.633 |
| MTx-%C ≤78 | 54.78 | 40.37 | 5.43 | 12.92 | 232.27 | <0.001 |
| Model for MTx-RT | | | | | | |
| Age (>60) | 0.56 | 0.34 | -0.96 | 0.17 | 1.84 | 0.337 |
| Education years (> 6) | 0.24 | 0.16 | -2.11 | 0.06 | 0.90 | 0.035 |
| Mental job | 0.72 | 0.40 | -0.59 | 0.24 | 2.14 | 0.558 |
| Sleep hours (>9) | 4.39 | 3.55 | 1.83 | 0.90 | 21.44 | 0.068 |
| Homocysteinemia | 2.02 | 1.38 | 1.03 | 0.53 | 7.72 | 0.303 |
| Number of cerebral infarctions (>1) | 2.25 | 1.48 | 1.23 | 0.62 | 8.16 | 0.217 |
| The differently affected regions of the AIS(Posterior) | 0.41 | 0.23 | -1.57 | 0.14 | 1.24 | 0.116 |
| MTx-RT >1.500 | 9.90 | 5.44 | 4.17 | 3.38 | 29.06 | <0.001 |
| Model for MTx-Cp | | | | | | |
| Age (>60) | 0.71 | 0.48 | -0.51 | 0.19 | 2.68 | 0.608 |
| Education years (> 6) | 0.28 | 0.20 | -1.75 | 0.07 | 1.17 | 0.081 |
| Mental job | 0.67 | 0.41 | -0.66 | 0.20 | 2.23 | 0.511 |
| Sleep hours (>9) | 3.26 | 2.87 | 1.34 | 0.58 | 18.33 | 0.181 |
| Homocysteinemia | 1.33 | 0.96 | 0.40 | 0.33 | 5.46 | 0.689 |
| Number of cerebral infarctions (>1) | 2.25 | 1.64 | 1.11 | 0.54 | 9.41 | 0.267 |
| The differently affected regions of the AIS(Posterior) | 0.39 | 0.24 | -1.51 | 0.11 | 1.33 | 0.132 |
| MTx-Cp ≤46.3 | 20.03 | 11.69 | 5.14 | 6.38 | 62.88 | <0.001 |

OR, odds ratio; SE, standard error; CI, confidential interval; MTx-%C, MemTrax percent correct; MTx-RT, MemTrax response time (seconds); MTx-Cp=MTx-%C/MTx-RT;

## Table 4. Baseline Information of Neuropsychological Assessments

|  | Overall(n=104) | Non-PSCI(n=44) | PSCI(n=60) | t or z or χ^2^ | p-value |
| --- | --- | --- | --- | --- | --- |
| baseline mRS |  |  |  |  |  |
| *Mean ± SD* | 0.96±0.72 | 1±0.75 | 0.93±0.71 | t=0.4628 | 0.645 |
| *Median（Q1-Q3）* | 1(0-1) | 1(0-2) | 1(0-1) | z=0.456 | 0.649 |
| *Min-Max* | 0-2 | 0-2 | 0-2 |  |  |
| baseline HAMA |  |  |  |  |  |
| *Mean ± SD* | 3.83±2.90 | 3.82±2.37 | 3.83±3.25 | t= -0.0262 | 0.979 |
| *Median（Q1-Q3）* | 3(1-6) | 4(2-5.25) | 3(1-7) | z=0.381 | 0.703 |
| *Min-Max* | 0-10 | 0-8 | 0-10 |  |  |
| baseline HAMD |  |  |  |  |  |
| *Mean ± SD* | 3.80±2.76 | 3.66±2.61 | 3.90±2.89 | t= -0.4380 | 0.662 |
| *Median（Q1-Q3）* | 3(2-6) | 3(2-6) | 3(2-5.25) | z= -0.345 | 0.730 |
| *Min-Max* | 0-10 | 0-8 | 0-10 |  |  |
| baseline MoCA score |  |  |  |  |  |
| *Mean ± SD* | 22.61±5.47 | 24.48±4.92 | 21.23±5.47 | t=3.1141 | 0.002 |
| *Median（Q1-Q3）* | 23(22-26) | 25.50 (23-28) | 22(21-24) |  |  |
| *Min-Max* | 0-30 | 6-30 | 0-30 |  |  |
| baseline NIHSS |  |  |  |  |  |
| *Mean ± SD* | 2.08±1.91 | 1.59±1.70 | 2.43±1.99 | t= -2.2612 | 0.026 |
| *Median（Q1-Q3）* | 2(1-3) | 1(0-2) | 2(1-3) | z= -2.411 | 0.016 |
| *Min-Max* | 0-8 | 0-7 | 0-8 |  |  |
| baseline BI |  |  |  |  |  |
| *Mean ± SD* | 85.48±13.04 | 86.82±12.76 | 84.50±13,27 | t=0.8945 | 0.373 |
| *Median（Q1-Q3）* | 90(80-90) | 90(80-95) | 90(80-90) |  |  |
| *Min-Max* | 40-100 | 50-100 | 40-100 |  |  |

## Table 5. Correlation Between MoCA and MTx

|  |  | r-value | 95%CI | p-value | remarks |
| --- | --- | --- | --- | --- | --- |
| Baseline MoCA | Baseline MTx correct | 0.4632 | 0.2972~0.6021 | <0.001 | Pearson |
| End MoCA | End MTx correct | 0.6058 | 0.4677~0.7149 | <0.001 | Pearson |
| MoCA change | MTx change | 0.5894 | NA | <0.001 | Spearman |
