# Supplementary material for: Using MemTrax memory test to screen for post-stroke cognitive impairment after ischemic stroke: a cross-sectional study
Source: Front Hum Neurosci. 2023 Jul 17;17:1195220. doi: 10.3389/fnhum.2023.1195220 (PMC10387538; doi:10.3389/fnhum.2023.1195220)

supplementary Fig.1-PSCI Diagnostic Flowchart

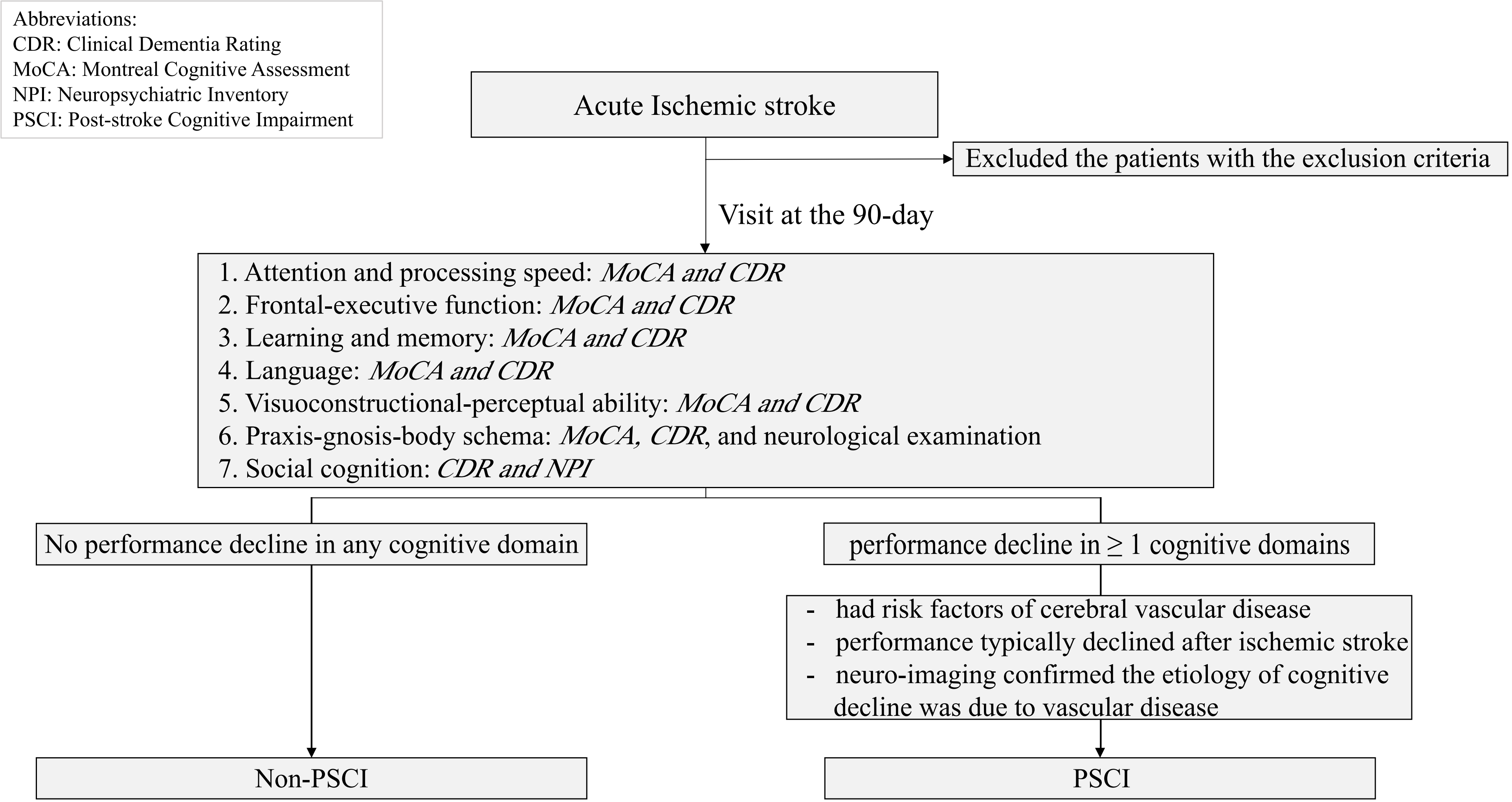

Supplement: Supplementary file 2 [file Image_1.pdf]
