# Supplementary figures and images for: Using MemTrax memory test to screen for post-stroke cognitive impairment after ischemic stroke: a cross-sectional study
Source: Front Hum Neurosci. 2023 Jul 17;17:1195220. doi: 10.3389/fnhum.2023.1195220 (PMC10387538; doi:10.3389/fnhum.2023.1195220)

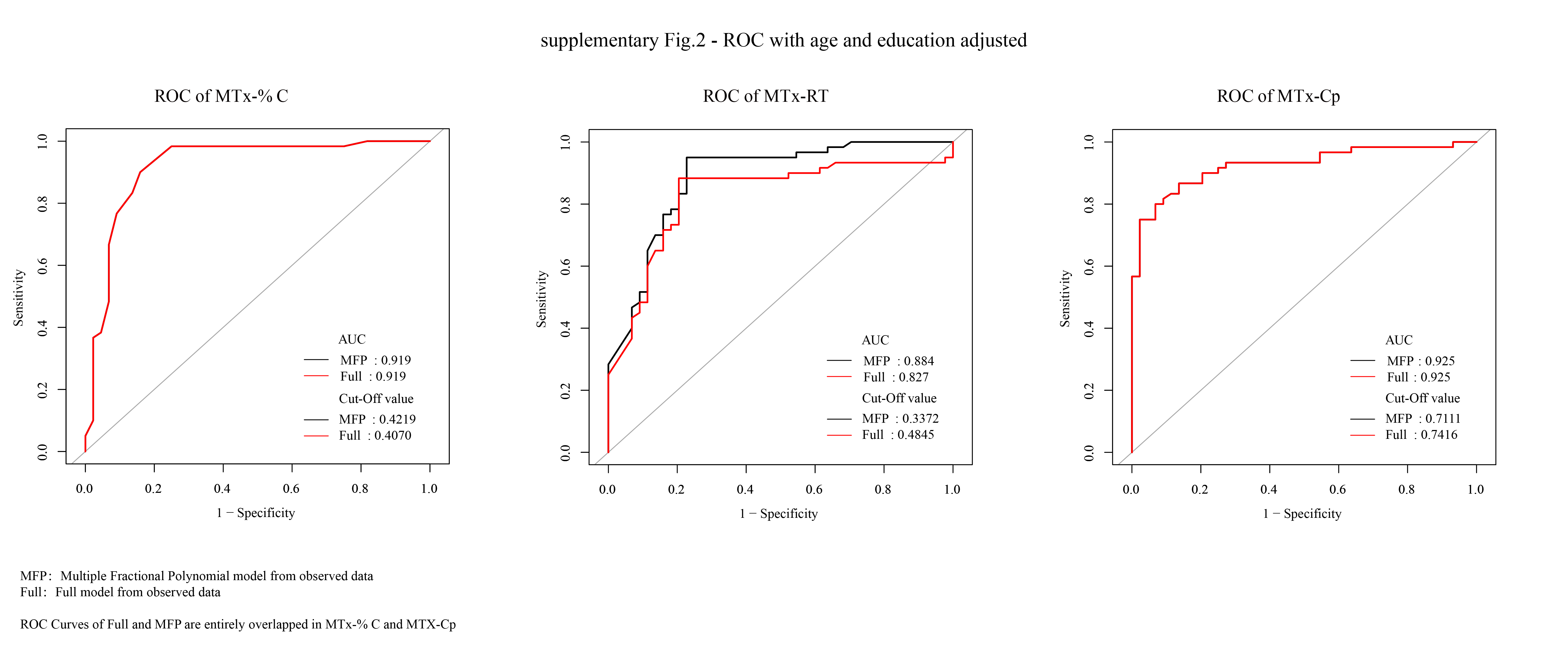

Supplement: Supplementary file 3 [file Image_1.TIF]
